# Bis(trimethyl­ammonium) tetra­chlorido­diphenyl­stannate(IV)

**DOI:** 10.1107/S1600536811000870

**Published:** 2011-01-15

**Authors:** Tidiane Diop, Libasse Diop, K. C. Kieran Molloy, Gabrielle Kocioc-Köhn

**Affiliations:** a Laboratoire de Chimie Minérale et Analytique, Département de Chimie, Faculté des Sciences et Techniques, Université Cheikh Anta Diop, Dakar, Senegal; b Department of Chemistry, University of Bath, Claverton Down, Bath BA2 7AY, England

## Abstract

The title compound, [(CH_3_)_3_NH]_2_[Sn(C_6_H_5_)_2_Cl_4_], consists of [(CH_3_)_3_NH]^+^ cations and [SnPh_2_Cl_4_]^2−^ anions in which the Sn atom, located on a centre of inversion, is bonded to four Cl atoms and two phenyl rings, giving an octa­hedral geometry with the phenyl rings in *trans* positions. In the crystal, the cations and the anions are connected by N—H⋯Cl hydrogen bonds and C—H⋯Cl inter­actions.

## Related literature

For background to organotin(IV) chemistry, see: Evans & Karpel (1985 ▶); Kapoor *et al.* (2005 ▶); Zhang *et al.* (2006 ▶). For compounds containing the [SnPh_2_Cl_4_]^2−^ ion in the *cis* or *trans* configuration, see: Ouyang *et al.* (1998 ▶); Hazell *et al.* (1998 ▶); Fernandez *et al.* (2002 ▶); Venkatraman *et al.* (2004 ▶); Garcia-Seijo *et al.* (2001 ▶); Casas *et al.* (1996 ▶); Teoh *et al.* (1992 ▶). For related crystal structures, see: Casas *et al.* (1996 ▶); Ouyang *et al.* (1998 ▶).
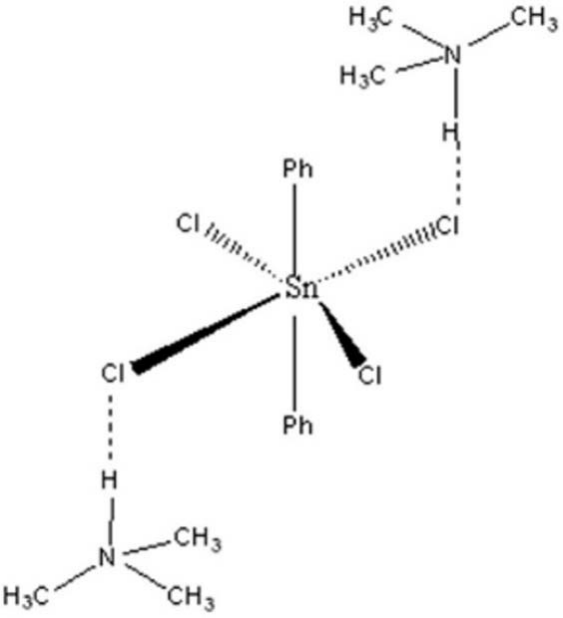


## Experimental

### 

#### Crystal data

(C_3_H_10_N)_2_[Sn(C_6_H_5_)_2_Cl_4_]
*M* *_r_* = 534.93
Monoclinic, 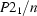

*a* = 9.0072 (2) Å
*b* = 8.4125 (2) Å
*c* = 14.9473 (4) Å
β = 96.046 (1)°
*V* = 1126.30 (5) Å^3^
*Z* = 2
Mo *K*α radiation
μ = 1.61 mm^−1^
*T* = 150 K
0.25 × 0.25 × 0.20 mm

#### Data collection

Nonius KappaCCD diffractometer
Absorption correction: multi-scan (*SORTAV*; Blessing, 1995 ▶) *T*_min_ = 0.689, *T*_max_ = 0.739
12905 measured reflections
2571 independent reflections
2219 reflections with *I* > 2σ(*I*)
*R*_int_ = 0.048

#### Refinement

*R*[*F* ^2^ > 2σ(*F* ^2^)] = 0.029
*wR*(*F* ^2^) = 0.072
*S* = 1.08
2571 reflections
122 parameters
H atoms treated by a mixture of independent and constrained refinement
Δρ_max_ = 2.36 e Å^−3^
Δρ_min_ = −1.41 e Å^−3^

### 

Data collection: *COLLECT* (Nonius, 1998 ▶); cell refinement: *SCALEPACK* (Otwinowski & Minor, 1997 ▶); data reduction: *DENZO*/*SCALEPACK* (Otwinowski & Minor, 1997 ▶); program(s) used to solve structure: *SIR97* (Altomare *et al.*, 1999 ▶); program(s) used to refine structure: *SHELXL97* (Sheldrick, 2008 ▶); molecular graphics: *ORTEP-3 for Windows* (Farrugia, 1997 ▶); software used to prepare material for publication: *WinGX* (Farrugia, 1999 ▶).

## Supplementary Material

Crystal structure: contains datablocks I, global. DOI: 10.1107/S1600536811000870/su2238sup1.cif

Structure factors: contains datablocks I. DOI: 10.1107/S1600536811000870/su2238Isup2.hkl

Additional supplementary materials:  crystallographic information; 3D view; checkCIF report

## Figures and Tables

**Table 1:** Hydrogen-bond geometry (Å, °)

*D*—H⋯*A*	*D*—H	H⋯*A*	*D*⋯*A*	*D*—H⋯*A*
N1—H1⋯Cl1	0.85 (3)	2.42 (3)	3.152 (2)	144 (3)
C5—H5⋯Cl1^i^	0.95	2.79	3.678 (3)	156

Symmetry code: (i) 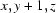.

**Fig. 1.**
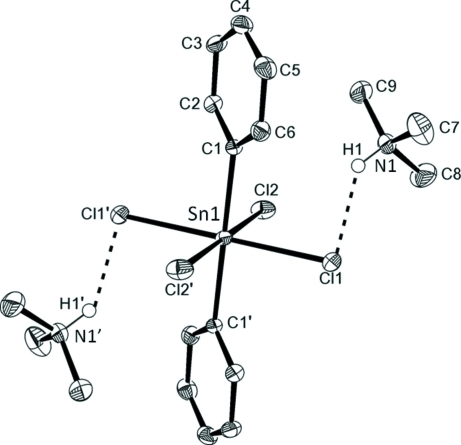
The molecular structure of the title compound with the atom-numbering scheme. Displacement ellipsoids are drawn at the 50% probability level [the C-bound H-atoms have been omitted for clarity; symmetry code: (') = -x + 1, -y + 1, -z + 1].

## supplementary crystallographic information

### Comment

Our interest for organotin(IV) compounds is related to the various applications found for this family of compounds (Evans & Karpel, 1985; Kapoor *et al.*, 2005; Zhang *et al.*, 2006). Many compounds containing the [SnPh_2_Cl_4_]^2-^ion in the *cis* or *trans* conformation have been reported (Ouyang *et al.*, 1998; Hazell *et al.*, 1998; Fernandez *et al.*, 2002; Venkatraman *et al.*, 2004; Garcia-Seijo *et al.*, 2001; Teoh *et al.*, 1992). In our search for new organotin(IV) compounds we have initiated here the study of the interactions between (CH_3_)_3_N.HCl and SnPh_2_Cl_2_, which has yielded the title compound.

In the [Ph_2_SnCl_4_]^2-^anion, the tin atom is located on a centre of inversion and is bonded to four Cl atoms and two phenyl groups giving an octahedral geometry with the phenyl groups in *trans-* positions (Fig. 1). Consequently, the angle between the two *trans* groups is exactly 180 ° while the phenyl rings are almost perpendicular to the equitorial SnCl~4~ plane [C1—Sn1—Cl1 = 89.39 (6)°, C1—Sn1—Cl2 = 90.86 (7)°]. The two Sn—C (phenyl) bond distances are 2.149 (3) Å. The Sn—Cl bond distances [2.5722 (6) and 2.5796 (6) Å] are similar to those reported for [Hthiamine][SnPh_2_Cl_4_]. H_2_O (Casas *et al.*, 1996), *i.e.* 2.573 (2) and 2.571 (2) Å. However, in 8-methoxyquinoliniumSnPh_2_Cl_4_ (Ouyang *et al.*, 1998) these two bond lengths are slightly different [2.5727 (8) and 2.6099 (8) Å].

In the crystal the anion and the cations are linked by N—H···Cl hydrogen bonds (Fig. 1) and C—H···Cl intermolecular interactions (Table 1).

### Experimental

The title compound was obtained as a white crystalline solid by reacting trimethylammonium chloride with diphenyltin dichloride in chloroform (2/1 ratio; *M*.p: 443 K). After slow evaporation of the solvent colourless crystals, suitable for X-ray diffraction analysis, were obtained.

### Refinement

The NH H-atom was located in a difference Fourier map and was freely refined. The C-bound H-atoms were included in calculated positions and treated as riding: C—H = 0.95 and 0.98 Å for CH and CH_3_ H-atoms, respectively, with *U*_iso_(H) = k × *U*_eq_(C), where k = 1.2 for CH H-atoms, and k = 1.5 for CH_3_ H-atoms.

### Crystal data

**Table:**

(C_3_H_10_N)_2_[Sn(C_6_H_5_)_2_Cl_4_]	*F*(000) = 540
*M**_r_* = 534.93	*D*_x_ = 1.577 Mg m^−^^3^
Monoclinic, *P*2_1_/*n*	Mo *K*α radiation, λ = 0.71073 Å
Hall symbol: -P 2yn	Cell parameters from 7261 reflections
*a* = 9.0072 (2) Å	θ = 2.9–27.5°
*b* = 8.4125 (2) Å	µ = 1.61 mm^−^^1^
*c* = 14.9473 (4) Å	*T* = 150 K
β = 96.046 (1)°	Block, colourless
*V* = 1126.30 (5) Å^3^	0.25 × 0.25 × 0.20 mm
*Z* = 2	

### Data collection

**Table:**

Nonius KappaCCD diffractometer	2571 independent reflections
Radiation source: fine-focus sealed tube	2219 reflections with *I* > 2σ(*I*)
graphite	*R*_int_ = 0.048
142 2.0 degree images with \v scans	θ_max_ = 27.5°, θ_min_ = 3.3°
Absorption correction: multi-scan (*SORTAV*; Blessing, 1995)	*h* = −11→11
*T*_min_ = 0.689, *T*_max_ = 0.739	*k* = −10→10
12905 measured reflections	*l* = −19→19

### Refinement

**Table:**

Refinement on *F*^2^	Primary atom site location: structure-invariant direct methods
Least-squares matrix: full	Secondary atom site location: difference Fourier map
*R*[*F*^2^ > 2σ(*F*^2^)] = 0.029	Hydrogen site location: inferred from neighbouring sites
*wR*(*F*^2^) = 0.072	H atoms treated by a mixture of independent and constrained refinement
*S* = 1.08	*w* = 1/[σ^2^(*F*_o_^2^) + (0.0339*P*)^2^ + 0.7419*P*] where *P* = (*F*_o_^2^ + 2*F*_c_^2^)/3
2571 reflections	(Δ/σ)_max_ < 0.001
122 parameters	Δρ_max_ = 2.36 e Å^−^^3^
0 restraints	Δρ_min_ = −1.41 e Å^−^^3^

### Special details

**Table:**

Experimental. multi-scan from symmetry-related measurements Sortav (Blessing 1995)
Geometry. Bond distances, angles *etc*. have been calculated using therounded fractional coordinates. All su's are estimatedfrom the variances of the (full) variance-covariance matrix.The cell e.s.d.'s are taken into account in the estimation ofdistances, angles and torsion angles
Refinement. Refinement of *F*^2^ against ALL reflections. The weighted *R*-factor *wR* and goodness of fit *S* are based on *F*^2^, conventional *R*-factors *R* are based on *F*, with *F* set to zero for negative *F*^2^. The threshold expression of *F*^2^ > σ(*F*^2^) is used only for calculating *R*-factors(gt) *etc*. and is not relevant to the choice of reflections for refinement. *R*-factors based on *F*^2^ are statistically about twice as large as those based on *F*, and *R*- factors based on ALL data will be even larger.

### Fractional atomic coordinates and isotropic or equivalent isotropic displacement parameters (Å^2^)

**Table:**

	*x*	*y*	*z*	*U*_iso_*/*U*_eq_	
Sn1	0.50000	0.50000	0.50000	0.0152 (1)	
Cl1	0.47692 (7)	0.31690 (7)	0.36079 (4)	0.0227 (2)	
Cl2	0.25333 (7)	0.63136 (8)	0.43640 (4)	0.0256 (2)	
C1	0.6252 (3)	0.6661 (3)	0.42855 (15)	0.0158 (7)	
C2	0.7359 (3)	0.6103 (3)	0.37812 (16)	0.0192 (7)	
C3	0.8137 (3)	0.7152 (3)	0.32797 (17)	0.0233 (8)	
C4	0.7807 (3)	0.8763 (3)	0.32840 (17)	0.0251 (8)	
C5	0.6730 (3)	0.9329 (3)	0.37967 (18)	0.0245 (8)	
C6	0.5941 (3)	0.8278 (3)	0.42938 (16)	0.0202 (7)	
N1	0.1432 (2)	0.2116 (3)	0.37059 (14)	0.0237 (7)	
C7	0.1802 (4)	0.0396 (4)	0.3674 (3)	0.0423 (10)	
C8	0.0931 (4)	0.2743 (4)	0.2800 (2)	0.0424 (10)	
C9	0.0323 (3)	0.2447 (4)	0.4350 (2)	0.0328 (9)	
H2	0.75850	0.50000	0.37790	0.0230*	
H3	0.88900	0.67660	0.29360	0.0280*	
H4	0.83220	0.94790	0.29340	0.0300*	
H5	0.65260	1.04370	0.38110	0.0290*	
H6	0.51900	0.86680	0.46380	0.0240*	
H1	0.224 (3)	0.258 (3)	0.391 (2)	0.022 (7)*	
H7A	0.09310	−0.01930	0.34000	0.0630*	
H7B	0.20770	0.00040	0.42870	0.0630*	
H7C	0.26400	0.02430	0.33160	0.0630*	
H8A	0.16890	0.25150	0.23930	0.0640*	
H8B	0.07840	0.38950	0.28350	0.0640*	
H8C	−0.00120	0.22340	0.25710	0.0640*	
H9A	0.01660	0.35970	0.43870	0.0490*	
H9B	0.06950	0.20380	0.49450	0.0490*	
H9C	−0.06240	0.19260	0.41420	0.0490*	

### Atomic displacement parameters (Å^2^)

**Table:**

	*U*^11^	*U*^22^	*U*^33^	*U*^12^	*U*^13^	*U*^23^
Sn1	0.0147 (1)	0.0171 (1)	0.0144 (1)	−0.0006 (1)	0.0041 (1)	0.0010 (1)
Cl1	0.0237 (3)	0.0258 (3)	0.0196 (3)	−0.0035 (2)	0.0072 (2)	−0.0058 (2)
Cl2	0.0181 (3)	0.0304 (3)	0.0286 (3)	0.0049 (2)	0.0043 (2)	0.0089 (3)
C1	0.0158 (11)	0.0185 (12)	0.0132 (11)	−0.0032 (9)	0.0025 (8)	0.0009 (8)
C2	0.0196 (12)	0.0212 (12)	0.0169 (12)	−0.0013 (9)	0.0030 (9)	0.0000 (9)
C3	0.0199 (12)	0.0335 (15)	0.0172 (12)	−0.0030 (10)	0.0056 (9)	0.0014 (10)
C4	0.0240 (13)	0.0290 (14)	0.0215 (13)	−0.0092 (11)	−0.0009 (10)	0.0070 (10)
C5	0.0287 (14)	0.0166 (12)	0.0275 (13)	−0.0034 (10)	−0.0007 (11)	0.0033 (10)
C6	0.0211 (12)	0.0191 (12)	0.0204 (12)	0.0005 (9)	0.0028 (9)	−0.0018 (9)
N1	0.0182 (11)	0.0313 (12)	0.0210 (11)	−0.0051 (9)	−0.0002 (9)	−0.0037 (9)
C7	0.0308 (16)	0.0350 (16)	0.060 (2)	−0.0007 (13)	−0.0005 (15)	−0.0132 (15)
C8	0.0371 (17)	0.068 (2)	0.0211 (15)	−0.0077 (15)	−0.0020 (13)	0.0081 (14)
C9	0.0277 (15)	0.0436 (17)	0.0282 (15)	−0.0044 (12)	0.0078 (12)	−0.0067 (12)

### Geometric parameters (Å, °)

**Table:**

Sn1—Cl1	2.5796 (6)	C5—C6	1.397 (4)
Sn1—Cl2	2.5722 (6)	C2—H2	0.9500
Sn1—C1	2.149 (3)	C3—H3	0.9500
Sn1—Cl1^i^	2.5796 (6)	C4—H4	0.9500
Sn1—Cl2^i^	2.5722 (6)	C5—H5	0.9500
Sn1—C1^i^	2.149 (3)	C6—H6	0.9500
N1—C9	1.485 (3)	C7—H7A	0.9800
N1—C7	1.487 (4)	C7—H7B	0.9800
N1—C8	1.479 (4)	C7—H7C	0.9800
N1—H1	0.85 (3)	C8—H8A	0.9800
C1—C6	1.389 (4)	C8—H8B	0.9800
C1—C2	1.393 (4)	C8—H8C	0.9800
C2—C3	1.394 (4)	C9—H9A	0.9800
C3—C4	1.388 (4)	C9—H9B	0.9800
C4—C5	1.383 (4)	C9—H9C	0.9800
			
Cl1—Sn1—Cl2	88.01 (2)	C1—C2—H2	120.00
Cl1—Sn1—C1	89.39 (6)	C3—C2—H2	120.00
Cl1—Sn1—Cl1^i^	180.00	C2—C3—H3	120.00
Cl1—Sn1—Cl2^i^	91.99 (2)	C4—C3—H3	120.00
Cl1—Sn1—C1^i^	90.61 (6)	C5—C4—H4	120.00
Cl2—Sn1—C1	90.86 (7)	C3—C4—H4	120.00
Cl1^i^—Sn1—Cl2	91.99 (2)	C4—C5—H5	120.00
Cl2—Sn1—Cl2^i^	180.00	C6—C5—H5	120.00
Cl2—Sn1—C1^i^	89.14 (7)	C5—C6—H6	120.00
Cl1^i^—Sn1—C1	90.61 (6)	C1—C6—H6	120.00
Cl2^i^—Sn1—C1	89.14 (7)	N1—C7—H7A	109.00
C1—Sn1—C1^i^	180.00	N1—C7—H7B	109.00
Cl1^i^—Sn1—Cl2^i^	88.01 (2)	N1—C7—H7C	110.00
Cl1^i^—Sn1—C1^i^	89.39 (6)	H7A—C7—H7B	109.00
Cl2^i^—Sn1—C1^i^	90.86 (7)	H7A—C7—H7C	110.00
C7—N1—C8	111.4 (3)	H7B—C7—H7C	109.00
C7—N1—C9	111.8 (2)	N1—C8—H8A	109.00
C8—N1—C9	111.4 (2)	N1—C8—H8B	109.00
C8—N1—H1	109.3 (19)	N1—C8—H8C	109.00
C9—N1—H1	106.8 (19)	H8A—C8—H8B	109.00
C7—N1—H1	105.7 (17)	H8A—C8—H8C	109.00
C2—C1—C6	119.4 (2)	H8B—C8—H8C	109.00
Sn1—C1—C2	119.52 (18)	N1—C9—H9A	109.00
Sn1—C1—C6	121.02 (19)	N1—C9—H9B	109.00
C1—C2—C3	120.4 (2)	N1—C9—H9C	109.00
C2—C3—C4	119.7 (2)	H9A—C9—H9B	109.00
C3—C4—C5	120.1 (2)	H9A—C9—H9C	109.00
C4—C5—C6	120.2 (2)	H9B—C9—H9C	110.00
C1—C6—C5	120.1 (2)		
			
Cl1—Sn1—C1—C2	−43.53 (19)	Sn1—C1—C2—C3	177.05 (19)
Cl1—Sn1—C1—C6	134.3 (2)	C6—C1—C2—C3	−0.8 (4)
Cl2—Sn1—C1—C2	−131.53 (19)	Sn1—C1—C6—C5	−177.54 (19)
Cl2—Sn1—C1—C6	46.3 (2)	C2—C1—C6—C5	0.3 (4)
Cl1^i^—Sn1—C1—C2	136.47 (19)	C1—C2—C3—C4	0.1 (4)
Cl1^i^—Sn1—C1—C6	−45.7 (2)	C2—C3—C4—C5	1.2 (4)
Cl2^i^—Sn1—C1—C2	48.47 (19)	C3—C4—C5—C6	−1.7 (4)
Cl2^i^—Sn1—C1—C6	−133.7 (2)	C4—C5—C6—C1	1.0 (4)

Symmetry codes: (i) −*x*+1, −*y*+1, −*z*+1.

### Hydrogen-bond geometry (Å, °)

**Table:**

*D*—H···*A*	*D*—H	H···*A*	*D*···*A*	*D*—H···*A*
N1—H1···Cl1	0.85 (3)	2.42 (3)	3.152 (2)	144 (3)
C5—H5···Cl1^ii^	0.95	2.79	3.678 (3)	156

Symmetry codes: (ii) *x*, *y*+1, *z*.
